# Memory and materiality: The becoming of biographic objects after war and forced displacement

**DOI:** 10.1177/13591835241275867

**Published:** 2024-08-28

**Authors:** Julia Sonnleitner

**Affiliations:** Department of Linguistics, 27258 University of Vienna, Vienna, Austria

**Keywords:** forced migration, Yugoslavia, object biography, affordance, social memory

## Abstract

The social life of things, in the aftermath of war and forced displacement, is associated with change in significance and value. Against a background of massive destruction and dispossession, object survival is exceptional. However, not every object that survives gains value equally. Private possessions that survive might not be attended to or be discarded. This complicates a straightforward coupling of person and surviving object. In this paper, the *becoming* of biographic objects is addressed. My interview partners fled the war in Yugoslavia in the 1990s as children. The objects they presented in biographic interviews have accompanied them throughout their lives. Rather than being mere prompts to tell life stories, these biographic objects, I suggest with Barad’s study, emerged in tandem with the biographic subject. By example of a wartime letter and a childhood object, I demonstrate how these things become biographic objects as they afford social action at various points in people's lives. My main argument is that things come to be biographic objects because they afford agency in specific socio-historic constellations.

## Introduction

Ever since Kopytoff's (1986) publication *The cultural biography of things*, the insight that objects have biographies as people do has sparked academic interest. At the core lies the recognition that things do not have one static meaning that remains stable but that there are shifts and ruptures in how things are perceived and used. However, the term ‘biography’ was critiqued for being associated too closely with human birth, life and death, whereas these might be difficult to tackle in objects (Hahn and Weiss, 2013; Ingold, 2021; Joy, 2009; Küchler, 2001; Saxer and Schorch, 2020). The main point of these critiques is that object biographies are not linear and predictable but make unforeseen jumps, mobilities, form unusual communities with other objects, are not attended to in certain periods of time and gain momentum at other times. According to this critique, the changes that things go through, be they spatial, temporal, perceptual, relational or affective, are contingent and cannot fully be predicted or reconstructed.

Indeed, war and forced displacement further complicate a linear understanding of object biographies. Wars entail an enormous scale of destruction not only of human lives but also of material culture. Cultural heritage, including archives, museums, galleries, registry offices and libraries are often under strategic attack by the aggressor. In the 1990s war in Yugoslavia, the destruction of heritage and private possessions was a purposeful strategy of warfare. Homes, containing people's personal possessions, were destroyed or abandoned and looted in due course. The few things that people were able to save might have been gradually lost, stolen or abandoned in the process of forced migration and asylum regimes (Bakondy and Širbegović, 2022; Hicks and Mallet, 2019; Khosravi, 2018; Üllen, 2016). The survival of pre-war possessions is therefore improbable and all the more remarkable.

This paper is about objects that survived and continuously changed their significance in the course of people's lives. My research partners fled the war in Ex-Yugoslavia in the 1990s when they were children and for most of their lives have lived in Austria. The surviving objects presented here are the outcome of biographic interviews, conducted for my research project [Language in Motion] (2020–2023) which centred on language and media, in particular the role of materiality for the lived experience of language. These objects would act as material foundation for the biographic accounts in the interview situation. Referring to Hoskins (1998), I call these items ‘biographic objects’. Hoskins observed in her field research among the Kodi in Sumba that life experiences were told and generated by the aid of objects. When people were asked to narrate their life stories, she found that there was no established genre for autobiographic narrative in this context. Only in connection to people's personal items, life experiences were told which Hoskins aimed to elicit. These objects, such as a personal betel bag, afforded biographic narrative and were so thoroughly penetrated with their owner's personality that they *became* the person.

Forced displacement makes us acutely aware that a biographic objects’ survival is improbable and therefore serendipitous. It complicates the relation between narrator and biographic object, shifting our focus to the gradual development of this relationship. As Dziuban and Stanczyk state: ‘The desire to preserve and protect is never a fully controlled and predictable affair’ (Dziuban and Stanczyk, 2020: 385). In a similar vein, Yi-Neumann (2022) pointed out that the very fact that objects survive war and forced displacement should not make us conclude that the relationship between a person and an object that is used to tell their life story is ‘natural’ or uncomplicated, as the very survival and re-interpretation of these items is never a straightforward process. Consequently, my aim is to reconstruct why things survive and emerged as survivor-objects in this research. By focussing on their itineraries (Hahn and Weiss, 2013), I argue that things come to be biographic objects because of the social action they have afforded both in the past and in the research situation. This will be illustrated by two objects that have survived the war and were used to voice biographic experiences. Based on these two examples, I will present the *becoming* of a survivor-object: the twists and turns at the junction of object and human biographies that become salient in retrospect.

In this paper, focus will be laid on the changing value that biographic objects have in people's lives. Although Hoskins understood objects to be props for biographic narrative (1998: 4), she nevertheless raises the important issue of agency in biographic accounts and in particular, agency's material dimension. Taking Hoskins’ sensibility for the material foundation of biographic narrative as a point of departure, I will argue with Barad (2003) that the biographic subject and the biographic object constitute each other. If biographies are fundamentally constructed and emerge in specific context-dependent situations, how is biographic work divided up between the person and the object? What do these objects afford at different points in time? In particular, moments will be highlighted when objects enter communication, when they mediate social action, and when agency is distributed between people and things (Gell, 1998; Strathern, 1988).

The underlying concept of memory in this paper is Maurice Halbwachs’ notion of collective memory (Halbwachs, 1985/1939): he states that there is no division between individual (private) and social (collective) memory. There is no boundary between the inner world of private memory or the outer world of collective memory as they are in constant exchange with each other. We can only remember in the form of discourses that are available to us because of our embedding within social groups (which he calls social milieus). Remembering is an intersubjective act, never a purely internal matter, and it is crucially mediated. Memory is intersubjective because it is construed in exchange with other subjects and because it is mediated through language, discourse and material culture. Contrary to understanding memory as a private, personal and internal affair, Halbwachs’ thoroughly mediated nature of memory is taken as a starting point for the central concern in this article: that the becoming of biographic objects can be explained with the agency they afford in social projects at various stages in people's lives. My intention in this paper is also to move away from interpreting biographic objects as expression of belonging and identification (e.g. Parrot, 2012, Bryant, 2014) and to explain why things have the significance they do due to what they afford in a particular moment in people's lives.

The paper begins by briefly reviewing the literature on biographic objects and forced displacement. ‘(Biographic) objects and forced displacement’ section introduces the research design and analytical lens, propounding the epistemological framework of why and how objects emerged in this research. In ‘Entangled agency: the emergence of biographic subject and object’ section, I substantiate my argument by highlighting two empirical examples: two biographic objects that were produced before and during the war, respectively, and have remained with the interview partners ever since. In the concluding section, I outline how these findings feed back to the discussion on biographic objects in the context of forced displacement, suggesting that agency is divided between subject and object as they are embedded in socio-historical environments.

## (Biographic) objects and forced displacement

Materiality in the aftermath of violence has been studied extensively. Navaro-Yashin (2009) describes people's emotional relation to objects that were abandoned or looted after the separation of Cyprus in 1974. Compelled to live with these left-behind objects, people expressed an atmosphere of melancholy that Navaro-Yashin captured with the concept of ruination. Hirsch (2012) stated that saved objects in the aftermath of the Holocaust, in particular photographs and other objects of aesthetic expression, become important mediational means of intergenerational transmission. For descendants of Holocaust survivors, who Hirsch terms the generation of post-memory, memory objects, that is, objects that prompt remembering, encompass desires and fantasies but at the same time evoke dread. According to Svašek (2014), objects in the afterlife of violence ‘gain particular meanings, appeal and emotional agency in specific social and political settings’ (2014: 139). She demonstrates how objects of personal remembrance are upscaled to collective memory, addressing larger audiences. By their transferral to museums and exhibition spaces, these objects create communities of memory, transmitting the past and, in some cases, inciting political action. Objects can also stand for seemingly opposing aspects like suffering *and* survival. Renshaw (2020), in her research on stolen objects as mnemonic devices in the aftermath of the Spanish Civil War, demonstrates that stolen things, in transmitted stories of survival, can become extremely agentive. Francoist perpetrators of mass killings thus retrospectively are interpreted as falling victim to the envy that their victims’ objects evoked and that led the perpetrators to murder and theft.

During the Yugoslav War in the 1990s, cultural heritage as well as people's personal possessions were systematically destroyed as a strategy of warfare (Halilovich, 2014, 2015). Survivors of the Bosnian genocide, ‘despite attempts to erase them physically, symbolically and bureaucratically’ (Halilovich, 2014: 236), were however able to reconstruct their own memory resources from few remaining memory objects, in on- and offline spaces. The reconstruction of these archives with the scarce resources that survived plays an important role in the ongoing restoration process. Surviving materiality (including a broad range of items such as video clips and DNA records^1^) are important mediational means for both personal remembrance, collective memory and its transmission, as well as forensic evidence of atrocities committed. Halilovich concludes: ‘[…] the social life of records, images and video clips can be as unpredictable and novel as the life of any living person’ (Halilovich, 2015: 90). In the context of war and displacement, therefore, what potentially becomes a biographic object is first and foremost a question of survival against a background of destruction and dispossession. The literature on surviving objects shows that these items gain a new significance after conflicts, mediate collective memory and might incite communal action.

However, not every object that survives becomes an object of personal significance. As Marschall (2019) demonstrates in her work on intra-African mobility to South Africa, many of her interview partners did not bring any memory objects from their country of origin and if they did, objects did not always have a mnemonic function, that is, were used to narrate biographic stories. In a similar vein, Turan (2010), in her research on the Palestinian diaspora and how they try to retain a ‘homeland’ through objects, reports a case where an inherited object (a teapot) of her interview partner's grandmother is stored away and is not imbued with affect because, as she argues, ‘it does not reflect a strong masculine and defiant character that he [i.e. her interview partner] is trying to express’ (Turan, 2010: 51). Turan argues that things become significant biographic objects because they enchant people. However, not every object that is inherited and could potentially become a biographic object has the power to enchant. Therefore, an important factor is an object's congruence with (gendered) life projects. Yi-Neumann (2022) critiques the heroic survivor-object plot in research on biographic objects and forced displacement. Comparing two objects that survived forced displacement from Syria, he shows how one of these objects, a hand-made teddy bear, is of central importance to the person. It is filled with memories and emotional attachment, whereas the other object, a metal bracelet, has no emotional significance to the owner who even considers giving it away. Yi-Neumann argues that the appreciation of these objects does not change in a linear way but is constantly adapted according to a perceptual horizon. These examples complicate a straightforward coupling of surviving object and (forced) migrant, that is, not every object that survives forced migration becomes a biographic object. The reason that certain things emerge as biographic objects therefore requires explanation.

## Entangled agency: the emergence of biographic subject and object

Until this point, I have discussed the precarious state of personal objects in the context of war and displacement. But even though things survive, they might not be particularly important to people. They might not enchant or affect them. As the context when these objects emerge (in the case of this research project as well as in many others) is the biographic interview, I will now turn to the particular situation of biographic production.

In biographic research, the focus shifted to contexts where biographic accounts are produced in everyday life or through academic intervention (Alheit and Dausien, 2009; Dausien, 2022) and, recently, to how these are enmeshed with the material world (Bettinger, 2021). Barad's notion of the apparatus has been productive for biographic research (Tamboukou, 2014) because it sheds light on the process whereby biographic subjects and objects are produced. The research setting – or, in Barad's terms, the ‘apparatus’ (2003: 816) – comprising such things as the research interest, methods, the research partners and researchers, discourses, materials, objects and (inter-)disciplinary discussions – creates the phenomenon that is observed. Barad, further elaborating Niels Bohr's reflections, states the causal relationship between observation practices and the outcome of specific phenomena. The reason why phenomena emerge in research is because of the practices that are employed to observe them. Apparatuses afford the emergence of both objects and subjects because they create what Barad calls the ‘agential cut’ (2003: 815): the intra-action that effects a separation between subject and object but only *within* a certain phenomenon. In other words, the separation between object and subject does not pre-exist the observation. Rather, agentive subjects and passive objects are an *effect* of observational techniques. ‘The primary ontological units are not “things” but phenomena – dynamic topological reconfigurings/entanglements/relationalities/(re)articulations. […] Agency is not an attribute but the ongoing reconfigurings of the world’ (Barad, 2003: 818). Agency thus emerges from a specific configuration of subject and object, created by the apparatus. Entities, also called *relata*, emerge within an observable phenomenon, they come about because of the relation that is established between them. Therefore, relata do not preexist the relation but are ontologically dependent on the relation. Seen in this light, object biographies and human biographies that emerge due to a certain research apparatus do not have a life independent of each other. This way, Hoskin's rather static and narrow understanding of biographic objects as ‘props’ or ‘storytelling devices’ (Hoskins, 1998: 4) can be approached in a novel way, as objects and subjects constituting each other.

## Research design^2^

In field research that was carried out over a year, my ethnographic starting point was centred around the human biography. Objects emerged through this research because of their significance in the lives of the people who told me their life stories but even though people's biographies were at the empirical centre, these stories are about object biographies as well. Far from being linear or complete, human and object biographies continuously intertwine in the biographic accounts, the one facilitating the speaking about the other. The fieldwork comprised several consecutive one-on-one interviews with my research partners. The first session was a semi-structured interview centring on the topics of languages and media. In the second interview, I presented my first rough analysis of the first interview and the academic concepts that led to it. In this session, interview partners added their thoughts or, in rare cases, corrected some interpretations which led to a more trustful relation to the research. The main part of the research process was a period of time when the research partners would reflect on specific topics or periods of time of their own choice. They were encouraged to browse through their private archives – to look into their storage spaces and forgotten places or to look at familiar and displayed objects through the lens of media and language biographies. Interview partners chose one to two months to reflect on topics of their biographies and in the last session, they presented the outcomes of their reflections. Biographic objects could be brought to the interview either physically or as pictures (i.e. photos of objects) or just the description. Most interview partners chose to describe the objects and to include them in a biographic narration.

The decision to let the interview partners choose their own biographic focus meant to share power over the research focus. Prior to the commencement of the study, it felt like a risky choice: what if people did not speak about war or the escape from war at all? What if they chose to focus on a very recent time in their biographies and only talked about work-related topics? However, as it turned out, the choice to let interview partners decide on their own biographic focus had the advantage precisely in sharing sovereignty with regard to the research focus. Firstly, interview partners had the power to decide whether they wanted to share difficult and potentially traumatising memories which was an ethical issue. Secondly, it helped building motivation to participate in the project and building trust that they would not be asked questions about periods of their lives they did not wish to answer. The data generated by this procedure are biographic narrations. The life stories were facilitated by objects and vice versa, the significance of these objects in people's lives would be articulated by embedding them within history and the life story. If we call Barad's ‘agential cut’ (2003) into mind, the research process afforded subject and object, due to their relation, to come into being.

## From private archive to exhibition: upscaling collective memory

The common feature of the biographic narrations connected to *objects* was that interview partners voiced topics that were hardly touched upon in the first interview (without objects). Namely, war, escape and difficult experiences were articulated in connection to the things they brought to the last interview session. Moreover, objects not only brought forth points to talk about biographical aspects of people's biographies in the situation of the interview but also in previous situations in their lives. The argument I wish to illustrate with the following analysis, therefore, is that things *become* biographic objects because they have afforded communicative agency both in previous times and at the moment of the interview. I draw on two empirical examples to flesh out how the biographic object and subject come into being through their relation to each other. Things become biographic objects not just because they survive a war but because they continuously mediate agency. In all interviews that were conducted, things gradually became biographic objects due to their affordances at various stages in life. These two examples were chosen because they illustrate this continuous but changing affordance that was also typically observed in other cases. Whereas the first case, a letter, illustrates the upscaling of memory processes from family communication to collective memory, the second example, baby shoes, demonstrates that the biographic object affords both remembering and silence.

The first example, a letter, was produced during the war when the family was separated and which, in a later period of its biography, obtained an unexpected new meaning. This example shows how the letter's affordance changes in the course of history, in particular, its upscaling from family communication to collective memory. The letter emerges as a biographic object because of its affordances in different stages of Melisa B.'s life.

Vignette:

In 2020, almost 30 years after the war's outbreak, there is an exhibition at the Vienna Municipal Library about war and forced migration from former Yugoslavia. On my visit, one of the displays attracts my attention: it is a letter, written in Bosnian in a child's handwriting, comprising script and drawings and addresses the writer's father. The writer, presumably a girl who has just learned how to write, assures her father that they are well and alive. I eventually meet the author, Melisa B. (a pseudonym), at one of the talks that are given at the exhibition by people who contributed objects from their private archives for display. Later, in the interview, she reflects on these childhood letters and how they were caused by the family's separation in the early 1990s: Melisa's father had already moved to Austria, whereas her mother, her siblings and herself were still in Bosnia, planning to join their father. In the war situation, severed phone cables made transnational phone calls impossible which is why the children regularly wrote letters to their father. Because Melisa had just learned how to write, she describes how she sometimes struggled with this task. The letters were written in Bosnia, were sent to Austria and thereafter preserved. They are stored in a private archive for decades until one day, one letter is taken out and displayed at the exhibition.

If, referring to Joy (2009), relationships facilitate the production of things and things afford relationships, the letters, at the point of their production, facilitate the continuation of kinship relations between family members during the time of war and separation. They facilitate care relations across distance (Hromadžić and Palmberger, 2018). In this interview passage, Melisa B. reflects on how writing letters for her as a child was challenging, however, it was the only possible way to communicate with their father, as other communicative infrastructures were disrupted due to war. In this passage, Melisa compares letter-writing to telephone calls. A phone call might have been an easier way to communicate with her father back then and wouldn’t have caused her so much effort. However in due course, the letters gain an unexpected affordance: because of their materiality, they outlasted time and space. A telephone call would not have been kept, stored and displayed but a letter, due to its materiality, has this potential.

The biography of these letters consists ‘of a series of connected jumps as the object becomes alive within certain clusters of social relationships and is inactive at other points in time and space, undergoing a series of different lives and deaths’ (Joy, 2009: 144). In other words, people's and objects’ lives do not follow a linear temporality but are highlighted and contracted in biographic narrative. The longest time period in the letter's biography does not receive much narrative space, namely its storage in the private archive. For decades, the letters are stored and might not or only occasionally be taken out and looked at. This is a time span where little seems to happen in the letters’ lives, nevertheless, the archive provides a necessary infrastructure that assures the letters’ survival. At this stage, the letters become family keepsakes that might afford remembering the time when they were produced: childhood, the situation of war and the family's separation. As items of a private archive, the letters mediate a biographic experience that is shared by all family members. After decades of storage however, one of these letters transits to a different space, the exhibition, and again changes its significance and reach.

The next stage in the letter's biography is its display at the exhibition in the Vienna Municipal Library. At this stage, the letter's addressees are a much broader public. By its transit from private archive to public display, it affords the upscaling of a family keepsake to collective memory. Even though Melisa was struggling to write these letters in her childhood, their materiality turns out to be an affordance at a later stage. The letter can be displayed in an exhibition and provokes discussions about war time experiences. The letter is involved in action and allows Melisa B. to raise awareness about war, displacement and forced migration for an audience who does not have any experience with these issues. The biographic object, the letter, in this context facilitates the dialogue about biographic episodes. Thus, the letter enhances Melisa B.'s agency (awareness-raising) and authorises her as a speaker. The exhibition creates a frame for both the biographic object and the biographic subject to emerge, in close relation to each other. The author is the cause for the letter and vice versa, the letter in this context is the cause for authorising Melisa B.'s biographic account.

However, agency arising from the human–object relation can only unfold because it is crucially embedded in an environment, that is, a specific spatio-temporal frame. Preceding the exhibition were years of political labour by migrant and minority groups, both nationally and internationally, who prepared the ground for the historisation and museification of migratory movements. In Vienna, there are several prominent examples for this process.^3^ Associations of migrant and minority groups, such as *Initiative Minderheiten* or *Arbeitskreis Archiv der Migration* are important actors in the effort to understand migration as part of a national history, a fact that has traditionally been denied in Austrian national discourses (Wodak et al., 2009). Up to now, national archives and museums have rejected hosting permanent archives of migration. As a result, memory objects have to be stored in private or semi-public spaces (like e.g. restaurants) which poses problems for their preservation. Moreover, in private archives, memory objects are framed as personal or family keepsakes or might be discarded as clutter. An archive of migration, as the *Arbeitskreis Archiv der Migration* argues, would be a place to display these memory objects to evidence a shared, collective memory of migration and make them part of national history (Akkılıç and Bratić, 2020). Even though an archive of migration has up to now been denied in national archives, the aforementioned exhibitions helped to reconfigure discourse and to prepare ground for further exhibitions to take place. These previous exhibitions acted as discursive events that facilitated infrastructure for something like a letter to be taken out of the private archive and be treated as proposition for historical discourses. It shows that the letter's significance does not only spring from *individual* agency but from a specific socio-historical constellation (discursive shifts, political labour, material infrastructures) that create an agential cut (Barad, 2003) which affords a biographic object and subject to emerge.

## Reveal and conceal: a biographic object's double index

The following item, leather baby shoes, was brought to the last interview as a central biographic object. Being the only object that this interview partner still possesses from his childhood, they afford both remembering and concealing the past. The shoes mediate the transmission of memory within the family and illustrate that objects might not only change their significance in time but might also signify distinct memories for different family members.

Vignette:

The object that Luka K. (a pseudonym) introduces in our last interview as a result of his biographic reflections are leather baby shoes. They are currently displayed in his son's room and started to captivate his attention when he became involved in this research project. They were made in a famous Yugoslav shoe factory in Vukovar where his mother used to work before the war. This factory produced the most popular shoe brand in Yugoslav times. She made these leather shoes in the factory herself for her new-born son, Luka. When Luka is about two years old, the war begins in Slavonia, where they live in a neighbouring town of Vukovar. The family decides to escape to Austria two years later. Their initial application for asylum is denied, the family is pushed back and this goes back and forth for some time, until they finally receive a permanent residence permit. During this precarious time on the move, where things constantly had to be packed and unpacked, kept and discarded, the only object that survived from Lukas K.'s childhood are these shoes. He recalls that when they finally settled in a small town in Austria, the shoes were displayed in their home in what he calls a family shrine. The shoes are exhibited together with a cap and a bronze figurine, representing Luka's mother and brother respectively. Currently, the shoes are displayed in his son's room: ‘Luka K.: And that, I don’t know why because they [the baby shoes] have always hung in the room of the little one but since I had the task to look at objects from a different point of view, they often caught my attention. This made me sad // Interviewer: Yes. // but mainly this, back then, she [Luka's mother] used to work in Vukovar, in Vukovar it was all difficult // I: Yes. // then these shoes and the whole situation, because I hardly know anything about my childhood. It is almost like a / family secret, I wouldn’t call it // I: Mhm. // but it is just not talked about’. (2m_3)^4^

The analysis focusses on two stages: the shoes’ production in a Yugoslav factory and secondly, its display at home after the family's permanent settlement in Austria. Why did these shoes, contrary to everything else that was lost, survive the war, the escape, the time of push-back and re-migration to Austria and all subsequent moves? Firstly, the shoes’ production and the preservation are explained with their significance for Luka K.'s mother. Secondly, as a memory object, the shoes afford both remembering and concealing the past.

In the interview, Luka K.'s information about the shoes and about his childhood appear relatively minimal: his mother worked in a famous shoe factory in Vukovar. Considering the limited information that Luka K. has about this object, it is worth looking more closely to the specific time and place of production. The shoes’ preservation and display mirror the significance his mother had for them. For her, the shoes might reflect her time as a Yugoslavian factory worker (in fact, one of the most prestigious factories (see Cvek et al., 2015; Hrvatska tehnička enciklopedija, 2023). Working in such a prestigious factory meant financial independence, fringe benefits, sociability and status for the women employed there (Bonfiglioli, 2018). The shoes, therefore, might have not only been a symbol of a lost era and time before the war, they also represented a stark contrast between the mother's previous status and esteem through work and the precarity and insecurity of both work and legal status after the family fled to Austria. The shoes’ production and survival can therefore be attributed first and foremost to Luka K.'s mother who produced them and, during the time of insecurity, preserved them due to her labour and effort.

With the transit from Yugoslavia to Austria, the shoes are gradually transformed into an object that affords both evidencing of and concealing a violent past. In the family's home, the shoes are put into a careful arrangement with two other objects, namely a cap (representing Luka's mother) and a bronze figurine (representing his brother). As a community, these objects refer to a different spatio-temporal configuration, Yugoslavia, that, at the time of settlement, became the past. These items mediate a collective memory of the people they represent and state a common experience of an era. The shoes, the figurine and the cap are placed in a prominent location that Luka K. calls the ‘family shrine’ (2m_3). Certain areas in the home, like the entrance area or the living room, are ‘complex staging grounds’ (Halvorson, 2020: 144) for the display of highly charged objects (see also Csikszentmihalyi and Rochberg-Halton, 2002; Fehervary, 2012; Miller, 2001; Pink, 2004). Such home displays might index hidden knowledge (Halvorson, 2020: 146), knowledge that is not immediately evident to an external onlooker. Baby shoes in and of themselves refer to childhood due to their size and shape (even though, in reality, it is likely that they have never been worn). What however is not obvious, and which might be termed a hidden index, is the shoes signifying the Yugoslav era and the experience of war, displacement and new settlement. The shoes’ affordance, therefore, is precisely their double index: that they both state and conceal the past.

The shoes both reveal and conceal the past because they are involved in action: firstly, they are not used as prompts to tell a story but are imbued with silence. Secondly, they are connected to a word that is used as a cipher for what is not spoken about, namely ‘Vukovar’, which stands for extreme violence. Communication involving memory objects of a violent past is often connected to silence. Silence is however not the opposite of communication but also has a communicative function and is meaningful (Jaworski, 1993). Silence might pragmatically maintain ambiguity, as in this case, where the shoes as a double index both refer to and conceal the past. A growing body of research in memory studies discusses silence not as the pathological opposite of speaking but as a form of embodied, affective transmission. Especially with regard to memories of a violent past, there has been a move away from silence as the breakdown of speech, indicating trauma. Instead of understanding silence as pathological, requiring therapeutic intervention, these authors (e.g. Hirsch and Spitzer, 2009; Hirsch, 2012; Kidron, 2009, 2021; Riaño-Alcalá and Baines, 2011) suggest that silence might be seen as a form of agency.

Moreover, a number of authors have argued that silence in the transmission of memory is performative and it is often mediated by objects (Hirsch, 2012; Kidron, 2012) which are connected to affective attunement (Gilje, 2016; Škrbić Alempijević and Potkonjak, 2016). Affect and emotion come to the fore by performance and interaction with these things. ‘[O]bjects function as vital repositories of the past via active performance and enactment of human–object relations that bring the past sensuously, viscerally and emotively alive for rememberers’ (Kidron, 2012: 4). Affect and emotion are relational (Ahmed, 2010) and allow us to recognise people's orientations towards objects in acts of communication, signalling their relative value and importance. Kidron (2012) suggests how one might account for memory objects, mediating both transmission and silence. In her research, she shows how children and grandchildren of Holocaust survivors recall practices surrounding personal memory objects (e.g. a box of pre-war photographs and toys, scars on a back, a spoon from Auschwitz). Through the practice that is connected to these objects, the emotions that they evoke, and sometimes the taboo to see or touch these objects, descendants learn about their significance. At the same time, these objects get discursively connected to historical experiences through a clue, such as ‘German work camp’, ‘Auschwitz’, ‘Holocaust’. In the case of this example, ‘Vukovar’ similarly operates as a cipher for extreme destruction and war crimes (which Luka K. expresses as ‘in Vukovar it was all difficult’). Citing this town evokes historical knowledge without having to explicitly spell it out. Thus, even though objects do not serve as prompts to speak about the past, descendants are able to associate these objects with historical discourses that are mediated on a larger scale through mass communication and transcend knowledge transmitted within the family. By naming the town and thus invoking war atrocities, Luka suggests the cause for the silence about this period of family memory and the cause for his personal feeling about this object: the feeling of both sadness and being captivated by it. If we understand silence not as the opposite of communication but as a communicative action, the shoes afford communicative agency in terms of remembering the past. In the interview as well, the shoes are a medium to articulate both a childhood in Yugoslavia, the experience of war and the silence about this time due to the violent events.

## Conclusion

This paper examined biographic objects in the context of forced displacement. In particular, I explored the relation of people who escaped from war in Yugoslavia in the 1990s when they were children to their biographic objects that emerged in the research. Prior research on biographic objects and forced displacement has shown that the survival of personal objects against a background of massive destruction and dispossession is improbable and remarkable. What has been emphasised in the literature is these objects’ increase in significance in the aftermath of war. However, another strand of the discussion has complicated the link between survivor-object and person because not every object that survives equally gains significance. Consequently, a central issue that needs to be explored in this context is the *becoming* of a biographic object.

My central argument is that things become biographic objects because they afford agency. The objects discussed in this paper (and this is also true for all other objects that appeared in my research) became biographic objects not simply because they symbolise something that remains stable but because they are involved in social action. In other words, what they *are* is what they *do* at different points in time. Taking Maurice Halbwachs’ (1985/1939) concept as theoretical basis, I pointed out the thoroughly social nature of memory which involves objects as mediational means in specific social settings.

The two cases discussed above indicate that these objects became important in the interview partners’ biographies because they afford social action at particular points in time, including the interview. The childhood letter, being the only possible means of long-distance communication at the time of war, afforded family communication between Yugoslavia and Austria. Due to its materiality, the letter could be kept in an archive and become a family keepsake. Decades later, it became an exhibition display and facilitated the formation of a community of memory. The baby shoes, too, were created due to a parent–child relationship but also expressed the relation and attachment of my interview partner's mother to her workplace in a prestigious Yugoslav factory. Through their prominent display in the home after the family's settlement in Austria, the shoes afforded the remembrance and transmission of a shared past and at the same time, silence about the family's wartime experiences.

The results of this research have wider implications for the study of memory and materiality, in particular the survival of objects in the aftermath of war and dispossession. I argue that the discussed items become biographic objects because they have afforded agency in social projects. Thus, if we ask *when* certain objects become relevant, to whom and through which socio-historical processes, it becomes apparent that people's and objects’ biographies get entangled when their relation affords social action. At the same time, agency is not to be conceived as human agency acting upon inert matter but one that is equally enabled and restricted by materiality. Furthermore, agency does not spring from the person–object relationship alone but from their embedding in social and material environments: in the above examples, conditions for agency to emerge were connected to political labour, previous discursive events, care work or social institutions like family.

Arguably, the specific objects that emerged in the course of this research were epistemologically based on people's biographies. The research apparatus was the locale where these biographic accounts were produced. Biographic objects therefore afforded social action in the interview situation as well. They facilitated communicative agency to talk about aspects of the past. The particular research constellation realised an agential cut: the biographic subject and the biographic object came into being because of their relation. It was from this point that both the person's and the object's biography were reconstructed. Future research might explore biographic objects from different epistemological starting points, such as the objects’ biographies or the transmission of memory objects throughout history, thus putting the object at the centre of ethnographic research.

## Notes

1. On forensics and DNA technologies in the context of the Yugoslavian War in the 1990s see also Stover and Peress (1998), Wagner (2008), and Young (2017).

2. All interview partners were informed orally and in written form about why the research is being conducted, that anonymity is assured, and how the data collected is being stored prior to the commencement of the study. Research partners were also informed about the research procedure, the nature of data generated and how these data were going to be used in the publishing of the research findings. All interview partners gave their written consent. All names in publications are anonymised. The research design was participatory with regard to the choice of biographic topics and biographic objects. Transparency of the interpretative frame was aimed at by discussing a rough analysis of the first interview with the research partners. Research partners were recruited by snowball sampling. The criteria for participation were a certain biographic background (the escape from war in Yugoslavia in the 1990s when they were children) and their current residence in Vienna and surroundings.

3. For example, the exhibition ‘*Gastarbajteri. 40 Jahre Arbeitsmigration*’ about labour mobility caused by the Recruitment Agreement between Austria and South European countries during the time of economic growth between 1955 and 1973, or the exhibition ‘*Migration sammeln*’ and ‘*Geteilte Geschichte. Viyana - Beč - Wien*’, all of which displayed personal keepsakes and material culture of migrants’ private and work life in combination with audio material at the Wien Museum.

4. Note on Transcription /    change of syntax //    activities of affirmation or attention from the interviewer that do not indicate a turn, e.g. //I: I see.//
